# *Clostridium difficile* infection in children – clinical, diagnostic and therapeutic aspects

**DOI:** 10.1186/1471-2334-13-S1-P46

**Published:** 2013-12-16

**Authors:** Elena Cristina Popescu, Rozina Iagăru, Monica Luminos, Dorina Duma, Adina Stăncescu, Anuța Bilaşco, Georgeta Constantinescu, Elena Gheorghe, Luminița Marin, Gheorghiță Jugulete, Anca Drăgănescu, Angelica Vişan, Camelia Kouris

**Affiliations:** 1 National Institute for Infectious Diseases “Prof. Dr. Matei Balş”, Bucharest, Romania; 2 Carol Davila University of Medicine and Pharmacy, Bucharest, Romania

## Background

*Clostridium difficile* is the most common cause of antimicrobial-associated diarrhea and is a common healthcare associated pathogen. While asymptomatic carriage remains high among infants, recent epidemiological surveillance has demonstrated a rise in the prevalence of *Clostridium difficile* infection in children.

## Methods

We present 4 cases of children admitted to the National Institute for Infectious Diseases “Prof. Dr. Matei Balş” in the last year with *Clostridium difficile* colitis; the children had ages between 2 and 12 years. Diagnosis of infection was based on the presence of three or more stools in a 24 hour period, isolation of the organism and detection of toxins A/B in a diarrheal stool specimen (stool culture and PCR assay). We studied the clinical aspects and evolution after treatment.

## Results

All children presented an important risk factor for *Clostridium difficile* infection: antimicrobial therapy prior to infection. Three children presented chronic pediatric comorbidities: one case of Ewing’s sarcoma; one case of gastroesophageal reflux disease, recent abdominal surgery for gastric volvulus, use of proton pump inhibitors; one case of milk protein allergy. The clinical signs and symptoms were: fever (3 cases), watery diarrhea (2 cases), bloody diarrhea (2 cases), cramping abdominal pain, vomiting, loss of appetite and malaise.

The children received antimicrobial therapy: oral metronidazole in 2 cases, oral vancomycin in 2 cases, supportive treatment and probiotics, with good outcome. All patients recovered without complications. Relapse occurred in the case with osteosarcoma, after cytostatic treatment and he was treated with oral vancomycin. Rifaximin prophylaxis associated to the next cytostatic treatment prevented another relapse.

## Conclusion

1. The diagnosis of *Clostridium difficile* colitis should be suspected in patients with diarrhea and associated risk factors, even in the pediatric population.

2. The increasing incidence of *Clostridium difficile* infection requires the implementation of appropriate policies for antibiotic use and further studies investigating optimal therapeutic and preventive strategies.

